# Experimental Studies of an Asymmetric Multi-Bolted Connection under Monotonic Loads

**DOI:** 10.3390/ma14092353

**Published:** 2021-05-01

**Authors:** Rafał Grzejda, Arkadiusz Parus, Konrad Kwiatkowski

**Affiliations:** Faculty of Mechanical Engineering and Mechatronics, West Pomeranian University of Technology in Szczecin, 70-310 Szczecin, Poland; arkadiusz.parus@zut.edu.pl (A.P.); konrad.kwiatkowski@zut.edu.pl (K.K.)

**Keywords:** multi-bolted connection, exploitation state, bolt force monitoring, resistance strain gauges

## Abstract

This article describes the experimental studies of a preloaded asymmetric multi-bolted connection in the exploitation state. The construction of two stands were introduced: for bolt calibration and for evaluating the bolt forces in a multi-bolted connection. The bolts were tightened in a specific optimal sequence, in three passes, monitoring the force values in the bolts using a calibrated strain gauge measuring system. The studies were conducted for the selected multi-bolted connection on an Instron 8850 testing machine. The measurement data were saved in MATLAB R2018b Simulink. The measurement results were analysed statistically and are presented via charts showing the distributions of the normalised values of the bolt forces as a function of the linearly increasing and decreasing exploitation loads. We show that the forces in individual bolts, after unloading the multi-bolted connection, change in relation to the initial values of their preload.

## 1. Introduction

Multi-bolted connections are one of the most commonly used nodes in engineering structures. Experimental research and various types of analyses on them are still undertaken by scientists [1,2,3,4].

In the case of multi-bolted connections, the presence of two states of load and deformation should be considered. The first is the initial tightening of the connection [5], and the second concerns the loads and deformations in the exploitation state of the connection. The results of experimental tests of the tightening process of the selected asymmetric multi-bolted connection were described in a previous study [6]. The studies of the connection subjected to monotonic loads presented in this article are an extension of that study.

In the last two decades, experimental studies of multi-bolted connections under exploitation loads were mainly conducted for connections characterised by geometric symmetry. The mechanical behaviour of high-strength bolts in T-stubs, based on the moment of distribution, was described by Bao et al. [7]. Tartaglia et al. [8] studied the behaviour of T-stubs with preloaded bolts under large deformations. Analyses of the deformation process of T-stub flanges with preloaded bolts have been completed by Efthymiou [9], De Matteis et al. [10], Šliseris et al. [11], and Yuan et al. [12]. Jaszak [13] described the results of experimental studies, wherein the use of an elastic serrated gasket in bolted flange connections provided a significant increase in the connection tightness compared with the tightness of the connection sealed with a standard gasket. Bolt force variations in a bolted flange connection, due to the bending moment, were investigated by Bouzid [14]. Ding et al. [15] studied the effect of the number and arrangement of stiffeners on the stress state of bolted flange connections subjected to tension. Jaszak and Adamek [16] measured the bolt forces in a flange connection for different types of gaskets. Hoang et al. [17] experimentally tested bolted flange connections in pipe structures subjected to various types of loads.

Research on the stiffness of the selected bolted lap connections has been conducted by Lim and Nethercot [18], Wuwer et al. [19], and Puchała et al. [20]. Davaran [21] investigated the elastic and inelastic buckling of the bolted double shear lap connections. The fretting fatigue phenomenon in bolted high-strength steel lap connections was studied by Hämäläinen and Björk [22]. The behaviour of double shear lap connections with injection bolts was analysed by Kolstein et al. [23].

Gordziej-Zagórowska et al. [24] determined the deformation forms and strains in the case of a truss system composed of bars with an open cross-section. The experiment-based reliability of multi-bolted structural connections in a steel lattice tower has been analysed by Szafran [25] and Szafran et al. [26].

Kawecki and Kozlowski [27] investigated the bolt forces distribution in multi-row bolted end-plate connections. There have also been many studies on the beam-to-column bolted connections aimed at drawing moment-rotation [28,29,30,31,32,33] or force–displacement curves [34,35] for monotonic loads.

The conducted review shows that, currently, the most frequently analysed are typical multi-bolted connections occurring in engineering structures such as: flange connections, lap connections, connections in truss nodes, or beam-to-column connections. As mentioned above, these are most often symmetrical systems. A small number of studies concerned more general cases, i.e., connections with arbitrary bolt arrangement. Usually they related to foundation [36] or composite [37] connections. Here, we studied a multi-bolted connection using geometric and load asymmetry. This gives the tests a sense of universality and makes the tests innovative compared with those described in the literature.

The most common method of assessing bolt forces is the resistance strain gauge method. It is one of the most accurate methods of this type [38]; therefore, it is also implemented in this article. The research described in this study can be used to verify the innovative method of modelling nonlinear asymmetric multi-bolted connections presented in [39,40].

The article is structured as follows: Section 2 describes the construction of the tested multi-bolted connection and discusses the issue of the calibration of bolts used in the connection. In Section 3, the main research stand and research procedure are described, including details of the bolt-tightening method and the method of loading the connection on the Instron 8850 testing machine. Section 4 focuses on the research findings and discussions. Section 5 contains conclusions from the conducted research.

## 2. Bolted Connection and Bolt Calibration

**Figure 1:**
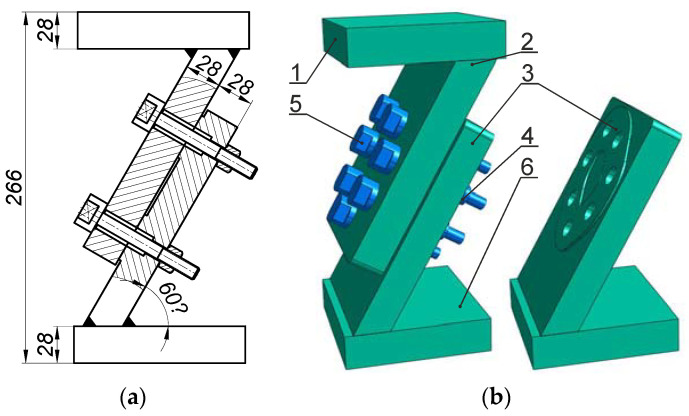
Tested connection: (**a**) diagram; (**b**) 3D view (1—upper plate; 2, 3—joined plates; 4—high hexagonal nut; 5—M10×1.25 bolt; 6—base).

In this study, we examined the multi-bolted connection that is presented in Figure 1. The tested connection consisted of two plates (2) and (3), fastened with seven special bolts with a thread profile of M10 × 1.25 (5) and high hexagonal nuts (4). The joined plates (2 and 3) were welded to the upper plate (1) and to the base (6). All joined elements were composed of steel S355J2 (1.0577). The bolts were fabricated in the 8.8 class of mechanical properties, and the nuts in class 8. To minimise the hysteresis during calibration, the bolts were heat-treated. Washers were not considered in the connection tests. A more detailed description of the multi-bolted connection can be found in [6].

**Figure 2:**
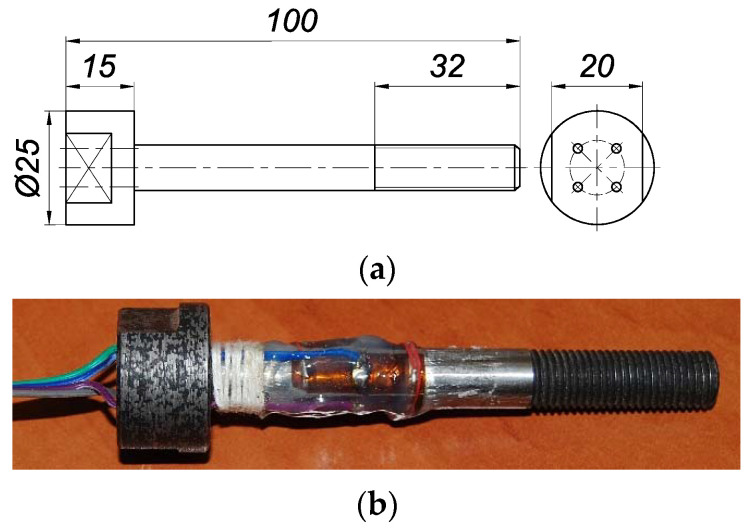
Single bolt used in tests: (**a**) diagram, (**b**) general view.

The diagram and view of the single bolt used in the research are shown in Figure 2. Changes in the force in each bolt were measured with four Tenmex TFxy-4/120 strain gauges. Each of them is characterised by the perpendicular arrangement of two axes of measuring ladders. The strain gauges were glued to the outside of the bolt shank in a full Wheatstone bridge configuration.

**Figure 3:**
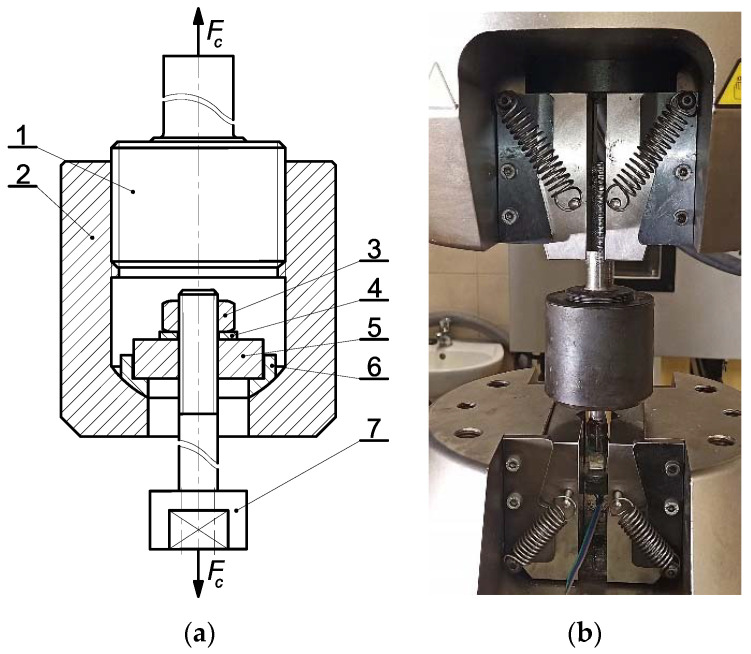
Equipment for calibration of the bolts: (**a**) diagram (1—special bolt, 2—handle, 3—nut, 4—washer, 5—plate, 6—spherical sleeve, 7—M10 × 1.25 bolt), (**b**) general view.

We began with the calibration of each bolt. For this task, the Instron 8850 testing machine was used, with additional equipment shown in Figure 3.

Each of the bolts (7) was placed successively in the spherical socket of the handle (2) using the plate (5) and spherical sleeve (6) set. The bolt was tightened with the normal hexagonal nut (3) in the class 10 of mechanical properties and the washer (4). Obtaining the ball joint this way ensured only the axial load of the bolt (7). The calibration was preceded by mounting the handle (2) through the special bolt (1) in the upper jaws of the testing machine and the head of the tested bolt (7) on its cylindrical surface in the lower jaws of the testing machine.

The results of the calibration are summarised in Table 1. They concern the regression equations for the processes of loading and unloading the bolts in the form [41]:

*F_ci_ = a_i_·V_i_*, (1)

where *F_ci_* denotes the calibration axial force of the *i*th bolt, *a_i_* is the slope of the regression curve, and *V_i_* denotes the voltage (for *i* = {1, 2, …, 7}).

All the slope factors of *a_i_* were obtained with the values of the determination coefficient *R*^2^ = 1. The obtained model characteristics of the bolts did not show hysteresis (they were identical for the processes of loading and unloading the bolts).

## 3. Main Research Stand and Research Procedure

**Figure 4:**
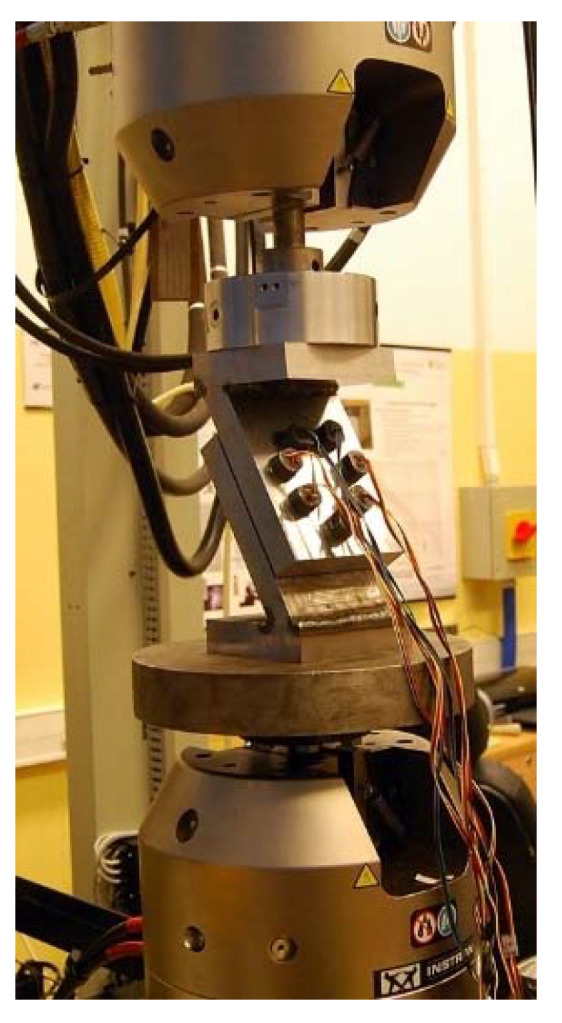
The main research stand.

The multi-bolted connection shown in Figure 1 was placed between the upper and lower heads of the Instron 8850 testing machine with the use of additional cylindrical support plates. The constructed main research stand is presented in Figure 4.

**Figure 5:**
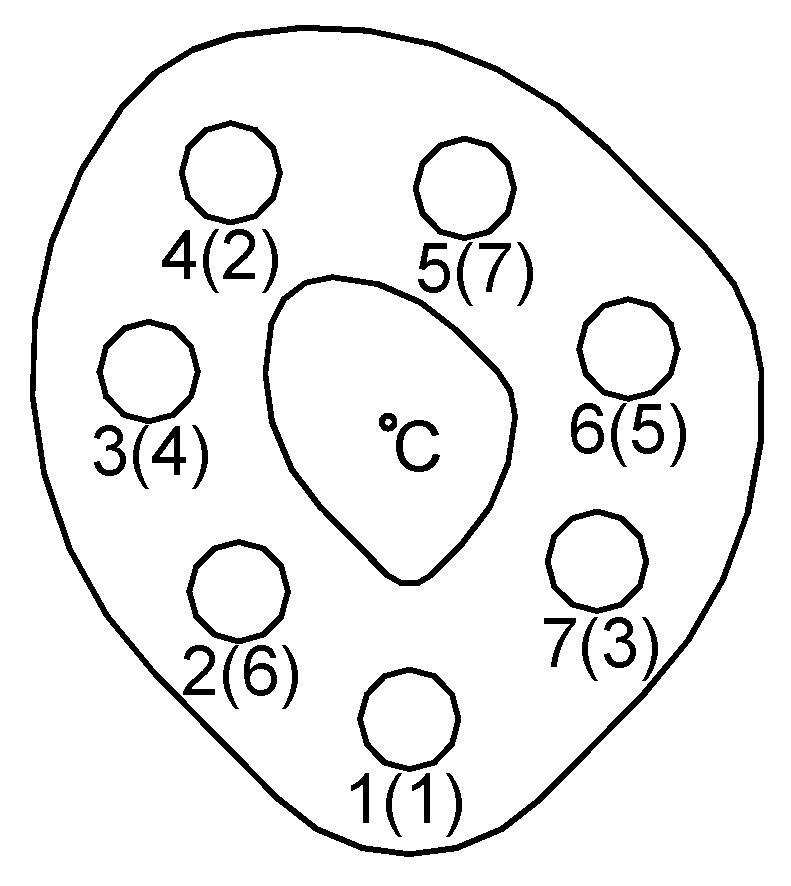
Location of the bolts in the plan of the contact surface between the joined elements.

The contact surface between the joined plates is presented in Figure 5. The area of this surface was irregular in order to created asymmetry in the tested connection. It fit in a circle with a diameter of 175 mm. The size of this area was equal to 89.2 cm^2^ and did not exceed the maximum pressure limit for steel S355J2 (1.0577). The bolts in the connection were located in accordance with the guidelines of the PN-EN 1993-1-8 standard [42].

The value of the bolts preload *F_p_* was assumed to be 22 kN on the basis of the PN-EN 1993-1-8 standard [42] and the maximum values of the pressure between the lower joined element and the nuts. The multi-bolted connection was tightened in three passes according to the order specified in [6] as the most optimal. The bolt tightening sequence is provided in parentheses next to the bolt numbers in Figure 5. In the first pass, the bolts were preloaded to the value of 0.2∙*F_p_*, whereas in the second and third pass, they were preloaded to the value of 0.6 *F_p_* and *F_p_*, respectively. During the exploitation state, the connection was monotonically loaded with the *F_e_* force with values from the set {0 kN, 10 kN, …, 40 kN, …, 10 kN, and 0 kN}. The maximum value of *F_e_* was set so that the shear forces caused by it did not exceed the friction forces on the contact surface between the elements joined in the connection. Each experiment was repeated three times. The next part of this article presents the values of bolt forces determined as the arithmetic mean of the data obtained in these experiments.

**Figure 6:**
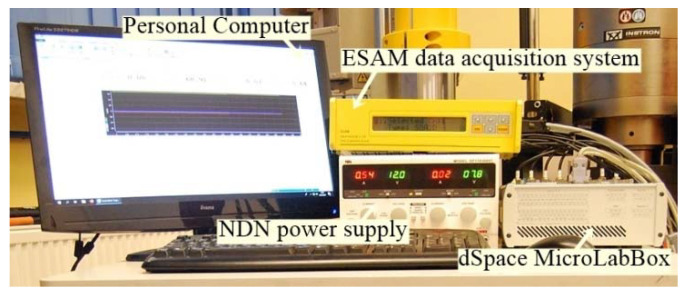
Bolt forces measurement set.

The reading and processing of experimental data were carried out using the measuring system, which is shown in Figure 6. The measurement data were saved in the MATLAB R2018b Simulink program.

## 4. Results and Discussion

**Figure 7:**
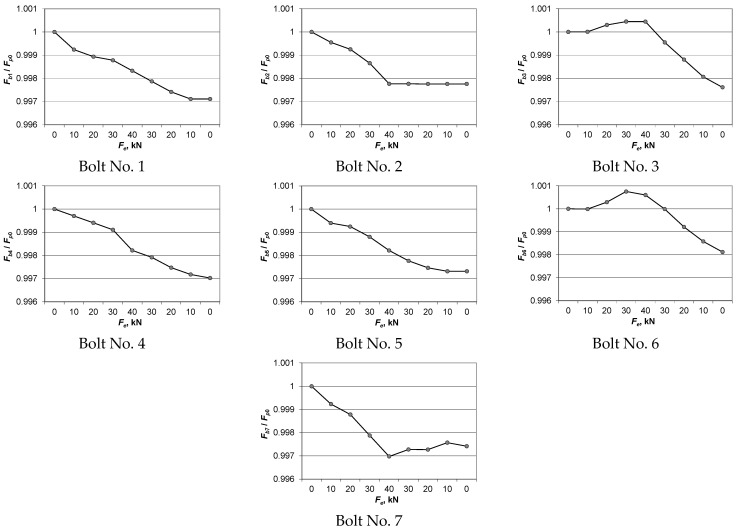
Distributions of the bolt forces as a function of the exploitation loads.

The distributions of the mean values of *F_bi_* in the bolts, referring to the initial force value *F_p_*_0_ during loading and unloading of the connection, are shown in Figure 7. As a result of the analysis of the charts in Figure 7, the following conclusions were drawn:

1. The bolt forces values varied upon the application of exploitation loads to a multi-bolted connection;
2. The variability in the bolt forces in the exploitation state of the multi-bolted connection depended on the bolt position in relation to the direction of the exploitation loads;
3. In the exploitation state, the bolt forces generally decreased. The exceptions were the forces in bolts No. 3 and 6, i.e., those lying near the straight line parallel to the connection base and passing through the centre of gravity of the cross-sectional areas of all bolts (point C in Figure 5);
4. In the unloading stage of the multi-bolted connection, the bolt forces generally decreased. The exceptions were the forces in bolts No. 2 and 7, i.e., those lying directly below the straight line parallel to the connection base and passing through the centre of gravity of the cross-sectional areas of all bolts (point C in Figure 5).

Multi-bolted connections are nonlinear systems of many bodies in contact. As a result, even in a preloaded multi-bolted connection, the load added in the exploitation state affects its stiffness characteristics and thus the change in bolt forces. When the connection is loaded, the unevenness in the contact joints occurring in the multi-bolted connection is levelled, so that the forces in the bolts are most often reduced. Unloading the connection further lessens these forces. The variability in the bolt forces is smaller in the case of multiple instances of loading and unloading of the connection. The results of research on this subject will be published in our next paper.

A quantitative comparative analysis of the charts shown in Figure 7 can be performed using the *Z*_1_, *Z*_2_, and *Z*_3_ indexes, which are defined as:

(2) $$Z_{1}=\frac{F_{p0}-F_{bi}^{40}}{F_{p0}}\cdot 100,$$

(3) $$Z_{2}=\frac{F_{bi}^{40}-F_{bi}^{0}}{F_{bi}^{40}}\cdot 100,$$

(4) $$Z_{3}=\frac{F_{p0}-F_{bi}^{0}}{F_{p0}}\cdot 100,$$

where $F_{p0}$ denotes the bolt force after the tightening process, $F_{bi}^{40}$ is the force in the *i*th bolt corresponding to an exploitation load of 40 kN, and $F_{bi}^{0}$ denotes the force in the *i*th bolt after unloading the multi-bolted connection.

The meaning of the indexes defined in Equations (2)–(4) is as follows:

- The *Z*_1_ index enables a relative evaluation of the changes in bolt forces during loading of the multi-bolted connection;
- The *Z*_2_ index enables a relative evaluation of the changes in bolt forces during unloading of the multi-bolted connection;
- The *Z*_3_ index enables a relative evaluation of the changes in bolt forces after the loading and unloading process of the multi-bolted connection in reference to the initial values of the preload in the bolts.

The values of the *Z*_1_, *Z*_2_, and *Z*_3_ indexes calculated for individual bolts are collated in Table 2. As a result of its analysis, the following conclusions were drawn:

1. Under the load of the multi-bolted connection with the *F_e_* force of 40 kN, the bolt forces decreased by 0.3%;
2. During the unloading of the connection, the bolt forces decreased by 0.28% in relation to their values corresponding to the maximum value of the exploitation load;
3. The forces in the bolts after unloading the connection decreased by 0.3% compared to the initial values of their preload.

Here, we studied the multi-bolted connection loaded in the exploitation state within the safe range for the connection structure. The variation in bolt forces with this approach is insignificant. The conclusion from this is that a properly selected value of the preload of bolts for a given exploitation case of the multi-bolted connection ensures its correct and safe functioning. However, we plan to perform similar tests with increased exploitation forces leading to the destruction of the connection. In our opinion, the variability in the bolt forces in this case will be greater.

## 5. Conclusions

This article presented an original laboratory stand for testing a selected asymmetric multi-bolted connection. The tests were conducted under the exploitation load conditions of the preloaded connection. The connection was loaded with forces that did not cause the loss of connection capacity. The variability in the bolt forces during both loading and unloading of the connection was demonstrated. The laboratory stand can be used for subsequent tests, for example, in the field of cyclic loading of the connection.

## Figures and Tables

**Table 1:** Results of the bolt calibration.

Bolt Number	Regression Equation
1	*F_c_*_1_ = 0.0245·*V*_1_
2	*F_c_*_2_ = 0.0242·*V*_2_
3	*F_c_*_3_ = 0.0239·*V*_3_
4	*F_c_*_4_ = 0.0244·*V*_4_
5	*F_c_*_5_ = 0.024·*V*_5_
6	*F_c_*_6_ = 0.024·*V*_6_
7	*F_c_*_7_ = 0.0237·*V*_7_

**Table 2:** *Z*_1_, *Z*_2_, and *Z*_3_ index values (%).

Bolt Number	*Z*_1_	*Z*_2_	*Z*_3_
1	0.17	0.12	0.29
2	0.22	0	0.22
3	−0.04	0.28	0.24
4	0.18	0.12	0.30
5	0.18	0.09	0.27
6	−0.06	0.25	0.19
7	0.30	−0.04	0.26

## Data Availability

Data available upon request from the corresponding author.
